# Asymmetric Dynamic Attunement of Speech and Gestures in the Construction of Children’s Understanding

**DOI:** 10.3389/fpsyg.2016.00473

**Published:** 2016-03-31

**Authors:** Lisette De Jonge-Hoekstra, Steffie Van der Steen, Paul Van Geert, Ralf F. A. Cox

**Affiliations:** ^1^Department of Developmental Psychology, Behavioural and Social Sciences, University of GroningenGroningen, Netherlands; ^2^Department of Special Needs Education and Youth Care, Behavioural and Social Sciences, University of GroningenGroningen, Netherlands

**Keywords:** recurrence analysis, synergies, children’s learning, microdevelopment, cognitive development, dynamic skill theory

## Abstract

As children learn they use their speech to express words and their hands to gesture. This study investigates the interplay between real-time gestures and speech as children construct cognitive understanding during a hands-on science task. 12 children (*M* = 6, *F* = 6) from Kindergarten (*n* = 5) and first grade (*n* = 7) participated in this study. Each verbal utterance and gesture during the task were coded, on a complexity scale derived from dynamic skill theory. To explore the interplay between speech and gestures, we applied a cross recurrence quantification analysis (CRQA) to the two coupled time series of the skill levels of verbalizations and gestures. The analysis focused on (1) the temporal relation between gestures and speech, (2) the relative strength and direction of the interaction between gestures and speech, (3) the relative strength and direction between gestures and speech for different levels of understanding, and (4) relations between CRQA measures and other child characteristics. The results show that older and younger children differ in the (temporal) asymmetry in the gestures–speech interaction. For younger children, the balance leans more toward gestures leading speech in time, while the balance leans more toward speech leading gestures for older children. Secondly, at the group level, speech attracts gestures in a more dynamically stable fashion than vice versa, and this asymmetry in gestures and speech extends to lower and higher understanding levels. Yet, for older children, the mutual coupling between gestures and speech is more dynamically stable regarding the higher understanding levels. Gestures and speech are more synchronized in time as children are older. A higher score on schools’ language tests is related to speech attracting gestures more rigidly and more asymmetry between gestures and speech, only for the less difficult understanding levels. A higher score on math or past science tasks is related to less asymmetry between gestures and speech. The picture that emerges from our analyses suggests that the relation between gestures, speech and cognition is more complex than previously thought. We suggest that temporal differences and asymmetry in influence between gestures and speech arise from simultaneous coordination of synergies.

## Introduction

How do children learn and develop understanding? How does cognitive change arise? In developmental psychology, this is one of the most intriguing questions, as evidenced by the considerable literature on the topic (see for instance, Piaget and Cook, 1952; Sternberg, 1984; Perry et al., 1988; Siegler, 1989; Carey and Spelke, 1994; Vygotsky, 1994; Thelen, 2000; Gelman, 2004; Anderson et al., 2012; Van der Steen et al., 2014). In search for the mechanisms behind cognitive development, the hands of children have come up as a vital ingredient. As children learn new things, or when they communicate or explain things, they use both their speech for verbal utterances and their hands to gesture (Goldin-Meadow et al., 1992; Anastas et al., 2011; Alibali and Nathan, 2012).

Gestures and speech are coupled, and mostly they are well-aligned, such that meaning expressed in gestures matches that expressed in speech. However, sometimes gestures and speech do not overlap, and a so-called gesture–speech mismatch occurs (Church and Goldin-Meadow, 1986; Perry et al., 1992; Goldin-Meadow, 2003). It has been demonstrated that during such gesture–speech mismatches, people (children and adults) express their cognitive understanding in gestures before they are able to put them into words (Crowder and Newman, 1993; Gershkoff-Stowe and Smith, 1997; Garber and Goldin-Meadow, 2002). Gesture–speech mismatches are especially likely to occur when a person is on the verge of learning something new. This makes them a hallmark of cognitive development (Perry et al., 1992; Goldin-Meadow, 2003), and shows that gestures and cognition are coupled as well. In the literature the explanation for this link has been attributed to gestures being a medium to express arising cognitive strategies (Goldin-Meadow et al., 1993), to highlight cognitively relevant aspects (Goldin-Meadow et al., 2012), to add action information to existing mental representations (Beilock and Goldin-Meadow, 2010), to simulate actions (Hostetter and Alibali, 2010), to decrease cognitive load during tasks (Goldin-Meadow et al., 2001) and to construct cognitive insight (Trudeau and Dixon, 2007; Stephen et al., 2009a,b;Boncoddo et al., 2010).

A conceptual framework which has been largely ignored in the research on gestures, and which follows from the work by Iverson and Thelen (1999), is that of synergetics and self-organization dynamics introduced by Haken (1977/1983), Kugler and Turvey (1987), and Kelso (1995). First of all, at the behavioral level, gestures and speech are considered to be action systems (Reed, 1982) That is, they are functional units organized to perform a specific task, like a hands-on science task in the present study. In addition, at the coordination level, we argue that gestures and speech form two coupled synergies. Within the context of action control, a synergy is a temporarily stable task-specific collective organization (Kelso, 1995), which emerges through self-organization out of a large set of underlying components distributed across body, brain, and environment.

To elaborate, gestures and speech require the precise coordination of many different muscles, joints, neurons, as well as related perceptual subsystems. Speech articulation, even for the simplest utterances, involves well over 70 muscles in the respiratory, laryngeal (‘voice box’) and pharyngeal (throat) systems as well as of the mouth, the tongue, etcetera (Galantucci et al., 2006; Turvey, 2007). Moreover, speech is highly attuned, for instance, to auditory information, but also to vision (needed for, e.g., interpersonal communication). Gesturing results from the coordinated contractions of 10s of muscles in the shoulder, upper arm, forearm, hand, and fingers of both upper limbs (Weiss and Flanders, 2004), and involves a tight informational link to proprioceptive as well as visual subsystems to stay attuned to the environment. Synergies for speech and gestures consist of several (overlapping) neural structures involved in information-motor couplings, across the central nervous system. Cognitive subsystems loosely associated with attention, memory and the planning of movements will play a role in gestures as well as in speech. Importantly, the gesture and speech synergies share several of these underlying components, and their recruitment will temporally overlap in any given task (cf. Wijnants et al., 2012a).

During communication or the expression of thoughts and ideas, the gesture and speech synergies synchronize to a high degree (McNeill, 1992). This synchronization reflects that the self-organizing process underlying the creation of both synergies is able to recruit the underlying components in the service of both gestures and speech adequately and synchronously. In fact, because of the tight coupling of the gesture and speech synergies, trying not to use either gestures or speech while communicating, or to desynchronize them, proves to be detrimental for the other (Goldin-Meadow et al., 2009). Moreover, Goldin-Meadow et al. (2001) found that if children or adults do not gesture -either by instruction or by choice- while they explain how they solved a mathematical problem, they perform worse on recalling a list of words or letters that they had to remember while they explained the mathematical problem. Goldin-Meadow et al. (2001, p. 521) conclude that “…gestures and speech form an integrated and, indeed, synergistic system in which effort expended in one modality can lighten the load on the system as a whole.”

From the perspective of synergetics and self-organization dynamics, the decline in performance if one only speaks but does not gesture should be related to suboptimal coordination of the gesture and speech synergies. More generally, when demands on the action systems increase, such as, for instance, in a novel or challenging task, the synergies become relatively less stable and less synchronized as compared to less challenging tasks. Novel and challenging tasks often have several new and (seemingly) conflicting task constraints. Since synergies are task specific, different task constraints lead to different collective organizations, competing for existence and the recruitment of (shared) components. Following Wijnants et al. (2012a), who studied synergetic control under conflicting task constraints in the context of a Fitts task, we reason that the gesture–speech mismatch in a novel task (Goldin-Meadow, 2003) resides in a less optimal simultaneous organization and coordination of the gesture and speech synergies. As a result, the usually tightly coupled synergies of gestures and speech dissociate, due to overlapping recruitment of the underlying components involved, resulting in the observable gesture–speech mismatch. Consequently, a gesture–speech mismatch can take different forms, such as instances in which gestures convey different content than speech, in which there are only gestures but no speech, and in which there is only speech but no gestures, similar to what Goldin-Meadow et al. (2001) found.

Most studies examining the gesture–speech mismatch have thus far focused on series of problem solving events in which, across different trials with some time in between, children are asked to solve a certain problem and explain their solution. These studies have focused on children’s solutions to, for instance, a series of mathematical equivalence problems (Alibali and Goldin-Meadow, 1993), Tower of Hanoi-problems (Garber and Goldin-Meadow, 2002), conservation tasks (Goldin-Meadow et al., 1993), and gear solving tasks (Boncoddo et al., 2010). From these studies, it appears that children show new problem solving strategies by means of gestures in earlier trials, to be followed by speech one or multiple trials later. A more detailed understanding of how such patterns of gestures and speech arise, and how this relates to our proposal of suboptimal coordination of synergies and cognitive development, requires a study of children’s verbal and non-verbal behaviors as they occur in real time (Pine et al., 2007), that is, during a task, considering their temporal order and coupling. The current study investigates the non-linear, dynamic interplay of children’s gestures and speech as they construct their cognitive understanding during a hands-on science task. Analysis tools will be employed which allow us to quantify the process of dynamic attunement between speech and gestures across all possible time scales during the task.

The current focus on the coupled dynamics of gestures and speech as it occurs in the moment and across time scales resonates with the relatively recent call for microgenetic studies to investigate the process (rather than just the outcome) of cognitive development (e.g., Grannot and Parziale, 2002; Siegler, 2006; Flynn et al., 2007; Van der Steen et al., 2012; Cox and Van Dijk, 2013). These microdevelopmental studies are exponents of the complex dynamical systems approach to behavior, cognition, and development (Van Geert, 1998, 2003, 2011; Smith and Thelen, 2003). This approach aims to infer the “why” and “how” of development (Thelen and Corbetta, 2002), using the language of complex dynamical systems: multi-causality, self-organization, variability, stability, non-linearity and so on, and the accompanying data-analytical tools.

To explain these terms in short, multi-causality pertains to the notion that development cannot be ascribed to one component or level of the developing system, but instead emerges from the continuous interaction of all the levels of the developing system (Thelen and Smith, 2007). Self-organization means that patterns and order emerge from the continuous interaction of all levels of the developing system, without external interference. Variability and stability follow from self-organization, as both variable and stable behavior occur within a developing system. For new stable behavior, i.e., new patterns, to emerge, a system typically displays variable behavior before settling in a new, more stable, pattern. Variability is thus a hallmark of developmental change. Moreover, this indicates that development is inherently non-linear, with periods of stable and variable behavior (Van Geert, 2008). Multicausality, self-organization and variability are also mechanisms that are apparent in our proposal that diverse components coordinate to form the synergies of gestures and speech, and that the dynamics within and between the synergies, under certain conditions, result in gesture–speech mismatches.

Dynamic skill theory is a theory of cognitive development encompassing dynamical system principles (Van Geert and Fischer, 2009). It provides a model that allows researchers to structurally investigate processes of cognitive development (Fischer, 1980; Fischer and Bidell, 2006). Dynamic skill theory states that the development of cognitive skills — defined as actions and thinking abilities, which includes verbalizations and gestures — proceeds through a series of hierarchically, ordered levels. That is, the development of cognitive skills follows a structure in which higher-order skills are constructed of a combination of skills at lower levels. According to dynamic skill theory, skills develop through a series of 10 levels, divided over three tiers, although not in a simple linear fashion (see below). The first tier is the sensorimotor tier, which consists of perceptions, actions and observable relations between these perceptions and actions. The second representational tier goes beyond the observable relations between actions and perceptions, although still restrained to concrete situations. The last tier, abstractions, includes non-concrete rules that apply in general (Schwartz and Fischer, 2004). Each tier consists of three levels, single sets, mappings (relations between single sets), and systems (relations between mappings).

In accordance with the notion of nested timescales, which implies that development occurs at different, though tightly interconnected timescales, the levels as distinguished by dynamic skill theory are applicable to both macro (long term) and micro (short term) development (Schwartz and Fischer, 2004; Fischer and Bidell, 2006). This means that people also go through these levels on the short-term time scale, for example during a new task, in a non-linear fashion, so that drops, spurts and stable periods in understanding occur (Van der Steen et al., 2012). This makes this theory particularly suitable for detailed, within-task dynamical analyses. Furthermore, dynamic skill theory provides a structure in which the concepts expressed in and constructed by gestures and speech can be compared, as it can be applied to both actions and verbalizations (Granott et al., 2002; Hoekstra, 2012). Lastly, dynamic skill theory’s model can grasp meaningful intra-individual variability on the short term timescale, by allowing for fluctuations in cognitive understanding during a single task, as well as the (sometimes differing) levels displayed by gestures and speech. This intra-individual variability has been linked to learning and transitioning to a higher (cognitive) level (Van Geert and Van Dijk, 2002; Yan and Fischer, 2002; Goldin-Meadow, 2003; Schwartz and Fischer, 2004; Van Geert and Steenbeek, 2005; Siegler, 2007). Although it has never been studied explicitly, understanding at the level of the sensorimotor tier might lead to a different interplay of gestures and speech, compared to understanding at the level of the representational tier.

As learning is an inherently non-linear process (Van Geert, 2008), and intra-individual variability in cognitive understanding and strategies is a hallmark of transitioning to more advanced levels, non-linear time-series methods are needed to investigate these processes. One such method is recurrence quantification analysis (RQA; Webber and Zbilut, 2005; Marwan et al., 2007). RQA originates from the study of natural systems, and has recently been applied to the study of human behavior and development (e.g., Shockley et al., 2002; Aßmann et al., 2007; Wijnants et al., 2009, 2012b). RQA is based on the detection and quantification of recurrent (i.e., repeatedly occurring) behavioral states, one of the most fundamental and important properties of dynamic systems. By using RQA and the notion of recurrence, measures of interest in a dynamic analysis of the behavior of a system, such as stability, regularity, and complexity can be retrieved from the time series. For a full overview of the RQA method, see the paper by Marwan et al. (2007), and for a useful guide to applying it see the chapter by Webber and Zbilut (2005).

A methodological advancement of RQA, cross-recurrence quantification analysis (CRQA; Zbilut et al., 1998; Shockley et al., 2002; Marwan et al., 2007) will be used in this paper to study the interplay of gestures and speech. With CRQA, the shared dynamics of two coupled systems, such as, for instance, parent-child dyads (Dale and Spivey, 2006; De Graag et al., 2012; Lichtwarck-Aschoff et al., 2012; Cox and Van Dijk, 2013), staff-client dyads (Reuzel et al., 2013, 2014) and adult dyads (Shockley et al., 2003; Richardson and Dale, 2005; Richardson D.C. et al., 2007; Richardson M.J. et al., 2007; Louwerse et al., 2012) can be studied. In CRQA, recurrence is generally defined as some match of behavioral state in the two systems under study. In RQA and CRQA alike, recurrence is not confined to states at exactly the same moment, but it is also noted when these particular matching states occur in the systems at either an earlier or later point in time, in fact across all possible time scales. These time scales range from the smallest time scale of the sample rate (seconds), to the duration of the entire observation. Linear tools fall short to fully capture the underlying dynamics of the cognitive system, which is fundamentally non-stationary and non-linear, as well as continuously attuning to a changing environment. Recurrences of system trajectories, on the other hand, can provide important clues as to the system from which they derive, in this case, the cognitive system (cf. Marwan and Webber, 2014).

To summarize, children’s use of gestures and speech is known to be informative about their cognitive capabilities, which change on a developmental time scale (Goldin-Meadow, 1998). As we have argued above, synergetic control and synergetic competition form a valuable explanatory framework for this research topic, which might lead to novel insights. As synergies are reflected in the dynamic organization of behavior (cf. Stephen et al., 2009b), we will analyze children’s gestures and speech as they construct understanding in real time. To this end, CRQA will be applied to the two time series of skill levels (based on dynamic skill theory) displayed in children’s gestures and speech, while they are working on an educational science task. The main research question of this study is: how is the leading role of gestures over speech in children’s cognitive change, as reported in previous studies, related to and reflective of an underlying dynamic interplay between gestures and speech during task performance? Research outcomes will pertain to the dynamic attunement of gestures and speech, focusing, for instance, on their temporal relation, leader-follower hierarchy, and asymmetric coupling. Furthermore, the dynamic interplay between gestures and speech during task performance will be related to age and more general measures of performance outside the task. Specific research questions, hypotheses, and their rationale will be given after a more detailed introduction of recurrence procedures and the derived measures of dynamic organization in the Section “Materials and Methods.”

## Materials and Methods

### Participants

For this study, the data of 12 Dutch children, six boys and six girls, were analyzed. The participants took part in a larger longitudinal project (see Van der Steen, 2014), and were on average 39.1 months old (*SD* = 3.8) at the start of the longitudinal data collection. In this larger study, children individually worked on scientific tasks about air pressure and gravity, under guided supervision of a researcher, in 4-months intervals. All children were recruited at their daycare centers or (pre)schools by asking their parents for a written consent. Parents were told about the nature of the study (children’s longitudinal development of scientific understanding), but not about the specific tasks that were administered. The study was approved by the ethical committee of the Psychology Department of the University of Groningen.

For the current study, we chose to analyze children’s (non)verbal behavior during an air pressure task administered at the sixth measurement (see below). We chose this task because the task protocol gradually builds up to a wrap-up question in which children are able to show their understanding of the task at that point. Our sample included five children from kindergarten (*M* = 57.2 months, *SD* = 2.2 months), and seven children from first grade (*M* = 69.4 months, *SD* = 4.4 months). **Table 1** gives an overview of characteristics of each child, including children’s early math- and language-scores on standardized tests from a national pupil-monitoring system that the children performed in kindergarten. These tests are administered twice a year to keep track of primary school children’s progress on the subjects math and (Dutch) language. For the Kindergarten tests, children are asked to count, classify objects and phrase words. Scores can range from 1 to 5, with 1 as the lowest and 5 as the highest attainable score. In addition, **Table 1** provides children’s average skill level score during the past five measurements, as measured in their verbalizations.

**Table 1:** Overview of characteristics of the 12 participating children.

Child	Grade	Age (months)	Math-score	Language-score	Average score past tasks
1	KG	58	5	–	2.65
2	KG	55	5	5	2.27
3	KG	60	2	3	0.77
4	KG	58	5	5	2.55
5	KG	55	5	4	2.45
6	1	64	4	5	2.31
7	1	64	5	5	2.56
8	1	69	4	4	2.42
9	1	76	4	4	2.27
10	1	69	3	3	1.98
11	1	73	4	4	2.75
12	1	71	5	5	2.79
*Mean*	*–*	*64.3*	*4.25*	*4.27*	*2.32*

### Procedure

During the task, researcher and child were involved in a natural hands-on teaching–learning interaction. An adaptive protocol was constructed, which guaranteed that all children were asked the basic questions reflecting the core building blocks of the task and the incorporated scientific concepts (see Van der Steen et al., 2012 for an excerpt of an interaction). At the same time, the protocol left enough space for children to take initiative and manipulate the material. The researcher started by showing the task material to the child, asking about its purpose and functioning. The child was then encouraged to explore the material, while the researcher asked questions, such as “What do you think we should use this for?” Furthermore, the researcher was allowed to provide guidance by asking follow-up questions, encouraging the child to try out his/her ideas using the material, and by summarizing the child’s findings or previous answers. The guidance never included statements indicating whether the child was right or wrong. We analyzed the interaction until the child answered a ‘wrap-up’ question (“After investigating all of this, can you now explain how this device works?”), after which the protocol prescribed the researcher to start with another topic. This part of the interaction (from the first question until the ‘wrap-up’ question) took 5–12 min (on average a little over 8 min). All interactions took place within children’s schools, always guided by the same researcher, and were recorded on video.

### Materials

**FIGURE 1:**
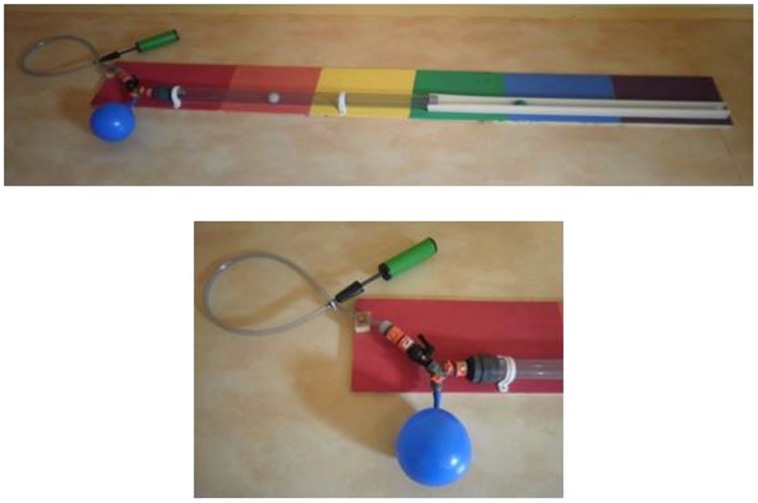
**The “air canon” and a close-up of the pump mechanism of this task**.

The task explored was called the “air canon,” specifically designed for this study. It was designed to let children explore how air pressure can be used to set materials in motion, and how air can be temporary stored in a balloon and released to have an even bigger impact on objects. The task consisted of wood, garden sprinkler parts, a transparent drainage tube, a gutter made from part of a room divider, a ball pump, balloon, and ping-pong balls (see **Figure 1**). There are three (sprinkler) taps on this device, one to (dis)connect the air pump, one to (dis)connect the balloon, and one to (dis)connect the drainage tube. Through questioning and exploring, children realize they have to open some taps (and close others) to make the canon work. There are two ways to shoot a ping-pong ball down the tube: (1) simply opening the taps connected to the pump and tube (closing the tap to the balloon), and repeatedly pumping, and (2) by inflating the balloon first (closing the tap to the tube), and then releasing the air into the tube. The colors on the wood serve as a measuring device to see how far the ball goes.

### Analysis

#### Coding Procedure

The interactions were first coded for children’s verbal utterances, and then for gestures/task manipulations. Both coding systems are described in more detail in the Appendix. The verbal utterances were coded in four steps using the computer program MediaCoder (Bos and Steenbeek, 2006). We started with the determination of the exact points in time when children’s utterances started and ended. The second step involved the classification of these verbal utterances into categories (e.g., description, prediction, explanation). As a third step, meaningful units of the child’s coherent task-related utterances were formed, so that utterances (sentences) about the same topic with only a short break in between were joined together for the fourth step. In this fourth and final step, the complexity of the child’s verbalized understanding within a unit was determined, using a scale based on Dynamic skill theory. The dynamic skill levels ranged from the levels of the sensorimotor tier to single abstractions, with levels of the representational tier in between. For example, at the first level of the sensorimotor tier (level 1), the child states a single characteristic of the task, such as “This tube is long.” At the first level of the abstract tier (level 7), the child mentions an abstraction that goes beyond the material, for example a statement about air pressure in general. This range of levels (1–7) approximately corresponds to the attainable levels for the children’s age (see Fischer and Bidell, 2006). Only utterances that displayed correct characteristics or possible task operations or mechanisms were coded as a skill level. This verbal coding procedure is explained in more detail elsewhere (Van der Steen et al., 2012, 2014).

In order to make sure that the codes of verbal utterances were reliable, a standardized codebook was used. For each step of coding, three raters went through a training of coding three video fragments of 15 min and compared their codes with those of an expert-rater (who constructed the codebook and training). The codes of the third fragment were compared to the codes of the expert-rater and a percentage of agreement was calculated. The reliability of the percentage of agreement is based on Monte Carlo permutation testing. The codes of one of the raters were shuﬄed 1000 times, so that the order of the codes became random. The *p*-value is the amount of times that the percentage of agreement of the shuﬄed codes was the same (or higher) as the empirical percentage of agreement, divided by the times that the codes were shuﬄed (1000). On average, the empirical percentage of agreement was: categories: 87% (range: 81–93; *p* < 0.01), combining verbalizations into units: 93% (range: 89–96; *p* < 0.01), and level of understanding: 90% (range: 83–95; *p* < 0.01).

The child’s gestures and task manipulations (hereafter: gestures) were coded independently from the verbal utterances. The coding procedure for gestures also involved multiple steps. During the first step, the exact point in time when a gesture started and ended was determined, along with a broad categorization of the gesture into the categories *short answers*, *representations/manipulations*, and *emblems* (such as “thumbs up”). For the second step, the broad categories of the first step were refined to more specific categories. For example, *short answers* were allocated to *nodding yes*, *shaking no*, etc., *representations/manipulations* were split into *characteristic* (such as representing ‘hard’), *movement* (such as representing ‘fast,’ or the course of a ball), *representation* (such as representing relations among different objects), while *emblems* were kept undifferentiated. The third and last step involved assigning levels of complexity, based on Dynamic skill theory (similar to how the verbal utterances were coded), to all *representations/manipulations*. For more details about the gesture codebook, see the Appendix, and Hoekstra (2012).

To ensure reliable coding of children’s gestures, two raters coded four training video fragments of 10 min independently, while following the standardized codebook, and their percentages of agreement were calculated for each step of coding. The reliability of the percentages of agreement was based on Monte Carlo permutation testing, like for the coding procedure for verbal utterances. On average, the percentages of agreement was: 97% (range: 94–100; *p* < 0.01) for the first step (broad categorization), 86% (range: 78–91; *p* < 0.01) for the second step (refined categories), and 92% (range: 88–98; *p* < 0.01) for the third step (level of complexity).

#### Time Series

**FIGURE 2:**
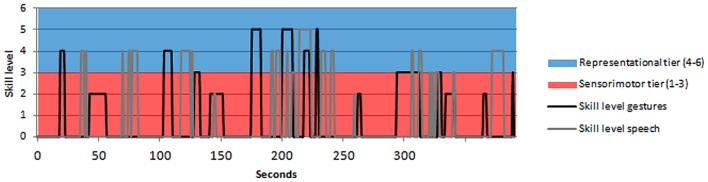
**Time series of skill levels of gestures and speech of one child (boy, 55 months)**.

Before performing CRQA on the data, the codes of the video fragments were transformed into a time series of the skill levels of speech, and a time series of the skill levels of gestures, with a sample rate of 1 s. If there was no event (i.e., no skill level), this was indicated with a 0 in the time series. In **Figure 2**, the time series of skill levels of gestures and skill levels of speech of one of the children in our sample is depicted. In order to be able to distinguish the lines in **Figure 2** clearly, only the first 300 s of the 392 s in total are displayed.

#### Cross Recurrence Quantification Analysis

**FIGURE 3:**
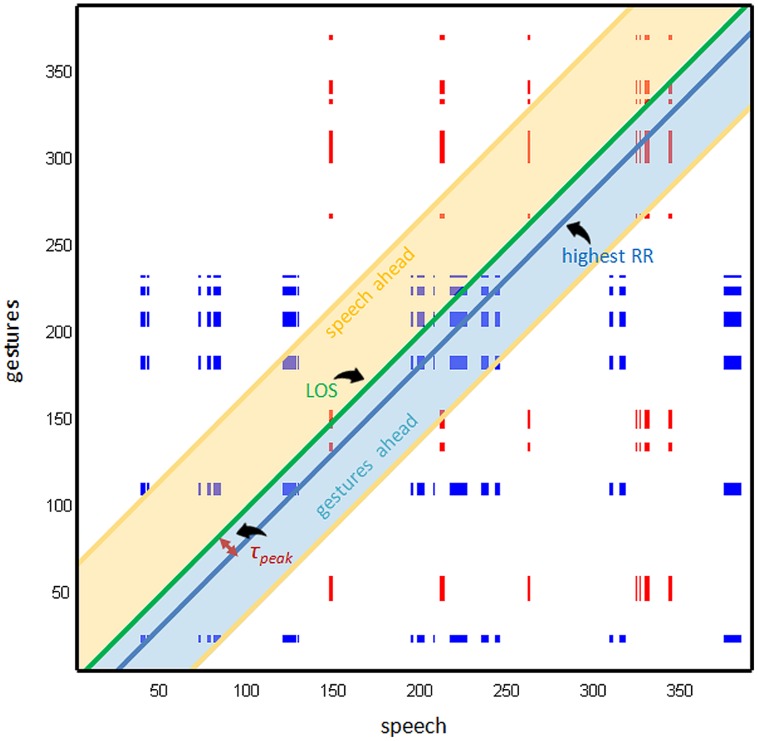
**Cross recurrence plot (CRP) of one child (boy, 55 months)**.

For categorical data, CRQA starts by plotting in a plane (called the cross recurrence plot, CRP, see **Figure 3**) all congruent appearances of some pre-specified matching values within a pair of time series, by putting one of the time series along the horizontal axis and the other along the vertical axis. Specifically, the CRP represents all those instances when the behavioral state of one subsystem (e.g., skill level in verbalization) at some moment in time is matched by the behavioral state of another subsystem (e.g., skill level in gesture) at the same or any other moment in time during the observation. These instances are depicted as colored dots in the CRP, which are canonically referred to as ‘recurrent points.’ From the spatial layout of these colored dots, several recurrence measures can be derived (see below). These CRQA-measures reveal hidden structure concealed in the shared dynamics of the two interaction subsystems (speech and gestures) across all possible time scales, which is informative about the dynamic organization of the cognitive system. **Figure 3** illustrates the CRP of gestures and speech for the same child as the time series in **Figure 2**. The CRPs of the other children are available as supplementary materials. In this study, matching states (i.e., recurrent points) are defined as same-tier skill levels, and are color-coded in the CRP as follows: blue dots represent instances in which gestures and speech both display a skill level from the sensorimotor tier (i.e., skill levels 1, 2, or 3). Red dots represent instances in which the skill levels as displayed by gestures and speech are both from the representational tier (i.e., skill levels 4, 5, or 6). Finally, yellow dots in the CRP represent a gesture–speech recurrence of the highest, abstract tier (i.e., skill level 7). The latter did not occur in our sample and these recurrences will therefore not appear in the analysis.

In **Figure 3**, the green diagonal line is the LOS, on which recurrent points have a delay of 0 s. These represent instances when both speech and gestures display a skill level from the same tier at the *exact* same time. The percentage of recurrent points on this line is called the percentage of synchrony (%Sync), which is a measure of linear static synchrony of the two subsystems. The recurrence rate (RR) is a measure depicting the proportion of recurrent points in the entire CRP. Hence, RR reflects the extent to which behaviors of one subsystem are matched by those of the other subsystem across all possible time scales, from the high end determined by the sample rate of 1 s, up until the low end determined by the duration of the observation. As such, RR is a basic measure of the coupling and coordination of the two subsystems. In the CRP of **Figure 3**, the skill-level time series of gestures is plotted on the vertical axis and the skill-level time series of verbalizations on the horizontal axis. This means that all colored dots above the LOS represent instances in which a skill level expressed in speech earlier in time is matched by same-tier skill level expressed in gestures at a later moment. Congruously, colored dots below the LOS represent instances in which skill levels from the same tier are displayed by gestures at an earlier moment and matched by speech later.

As can be seen in **Figure 3**, most colored dots in the CRP align to form block and line structures. Generally, such structures indicate instances where behaviors which are briefly expressed by one subsystem are accompanied by episodes of lingering in the matching behavior by the other subsystem. This provides information about the shared dynamics of the gesture–speech interaction, and specifically about the strength and direction of the coupling between the two subsystems, as we shall demonstrate (see Cox et al., 2016). Thus far, research using CRQA has focused on diagonal and vertical lines. However, notice how the line structures in the CRP stretch into the horizontal and vertical direction (and not diagonal), which is quite common for categorical time series. Analysis of the diagonal lines and the associated measures will therefore not be discussed here.

The different directions of the line structures (vertical and horizontal) provide differential and complementary information about the coupling between the two subsystems represented by the time series along the axes. For instance, a vertical line structure in the CRP (**Figure 3**) means that a brief skill-level expression in speech is followed (above LOS) or preceded (below LOS), with some delay, by a much longer same-tier skill level expression in gestures. Similarly, horizontal line structures represent instances in which a skill level that is expressed briefly in gestures, is followed (below LOS) or preceded (above LOS) by a much longer same-tier skill level in speech. More generally, line structures represent instances in which shortly expressed skill levels from a certain tier in one subsystem ‘trapped’ the other subsystem in a lingering same-tier expression for some time. In this study we will relate them to the relative strength and direction of the gesture–speech coupling, such that vertical line structures reflect the extent to which speech subsystems influence gestures, whereas horizontal line structures reflect the extent to which gestures subsystems influence speech.

To capture the asymmetric dynamic attunement between gestures and speech, we performed *anisotropic* CRQA (Cox et al., 2016), by calculating recurrence measures for the horizontal and vertical line structures separately and comparing them. The first measure derived from the line structures is ‘Laminarity,’ defined as the proportion of recurrent points that are part of a vertical (LAM_V_) or horizontal (LAM_H_) line structure. Laminarity reflects the degree to which subsystems are trapped into expressing a same-tier skill level for some period of time. LAM_V_ depicts how much gestures constitute larger structures of points in the CRP, whereas LAM_H_ does so for speech. Second, ‘Trapping Time’ is the average length of either the vertical (TT_V_) or horizontal (TT_H_) line structures. TT is measured in units of time and estimates how long subsystems are, on average, trapped in a specific state. In our study, the higher TT is, the longer a same-tier skill level from one time series lingers in the other one. If TT_V_ is high, gestures tend to be trapped in relatively long periods of same-tier skill levels that are also expressed by speech at some point, and for high TT_H_ speech tends to be trapped in relatively long periods of same-tier skill levels that are also expressed by gestures at some point. Finally, ‘Maximum Line’ also gives information about duration of line structures, with MaxL_V_ the length of the longest vertical line and MaxL_H_ the length of the longest horizontal line. In other words, MaxL measures the duration of the longest same-tier skill-level expression for speech and gestures. High MaxL_V_ means that gestures are trapped in a single tier of skill levels, and MaxL_H_ means that speech is trapped strongly in a single tier.

These three measures have been related to behavioral rigidity and regularity in previous studies (De Graag et al., 2012; Cox and Van Dijk, 2013). Accordingly, in the present study, we will interpret the CRQA-measures of horizontal and vertical line structures as ‘differential’ rigidity of speech and gestures, respectively. In addition, the relative size of these measures informs about the relative strength and direction of the coupling between speech and gestures.

#### LOS-Profile Analysis

**FIGURE 4:**
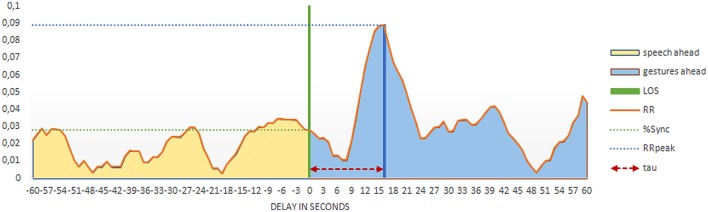
**LOS (line of synchrony) profile plot of one child (boy, 55 months)**.

Besides analyzing the global structure of the recurrence plot, we will also look in more detail at several recurrence measures within a smaller time window around the LOS (see, e.g., Richardson and Dale, 2005; Reuzel et al., 2013, 2014). **Figure 4** depicts the so-called LOS profile of an interval of 60 s on each side of the LOS, derived from the CRP in **Figure 3**. The LOS profiles of the other children are available as supplementary materials. The interval of 60 s above and below the LOS is chosen intuitively, so as speech and gestures can either lead or follow each other with a maximum delay of 1 min. In **Figure 4**, the position of the LOS, corresponding to a delay of 0 s, is indicated with a green line. The LOS profile is drawn ‘from the perspective’ of gestures, in that a positive delay indicates instances of recurrence in which gestures are ahead of speech in time (blue area), whereas a negative delay indicates instances in which speech is ahead of gestures (yellow area). The orange envelope curve represents the RR at each delay; this delay is called τ (RR_τ_ ; see, e.g., Marwan et al., 2007).

Several measures can be derived from this LOS profile, which inform about the coordination of the two subsystems within the chosen interval of 2 min around the LOS. Firstly, in **Figure 4** the RR shows a clear peak of around 0.09 at a delay of 16 s. This maximum recurrence rate, defined as the highest proportion of recurrent points within the LOS profile, is called RR_peak_, and is indicated with the blue line in **Figure 4**. The distance of this peak from the LOS (in seconds), or in other words, the delay of RR_peak_, is called *τ*_peak_, and is indicated with the red arrows. Please note that *τ*_peak_, with a value of 16 s, is also visible in **Figure 2**, as the skill levels displayed in gestures are clearly ahead in time of the skill levels displayed in speech. An example of what a match between gestures and speech with a delay of 16 s could be is: with his hands, a boy depicts that if you turn a switch, the ball will roll down the tube (level 3, tier 1). Around 16 s later, he says: “It [the ball] rolls, because it is round” (level 3, tier 1). The final measure that we can derive from the LOS profile is Q_LOS_. Q_LOS_ is the total proportion of recurrent points at the left side of the LOS (yellow area), divided by the total proportion of recurrent points at the right side of the LOS (blue area). If Q_LOS_ is lower than 1, this indicates that gestures are generally leading speech in time, whereas a Q_LOS_ with a value higher than 1 indicates the opposite.

### Research Questions and Hypotheses

The research question of the current study is: does the leading role of gestures over speech in children’s cognitive change, as reported in previous studies, arise from and reflect an underlying dynamic interplay between gestures and speech during task performance? To answer this general question, four specific research CRQA questions and corresponding hypotheses were formulated, which will be introduced below.

#### Research Question 1

The first research question is: what is the temporal relation between gestures and speech, with regard to the displayed (skill) level of understanding? Studies thus far demonstrated that, across tasks, children express their cognitive insights in gestures before they are able to put them into words (Crowder and Newman, 1993; Gershkoff-Stowe and Smith, 1997; Garber and Goldin-Meadow, 2002). Here we will investigate whether these results can be extrapolated to a smaller (i.e., within-task) time scale, and whether theoretical claims of previous studies can be corroborated and possibly extended to the perspective of gesture–speech mismatches as originating from the suboptimal simultaneous coordination of the gestures- and speech synergies. To this end we performed LOS-profile analysis on the gesture–speech interaction. The associated measures should display a significant asymmetry in the amount of recurrence around the LOS (Q_LOS_) and display a recurrence peak (RR_peak_) at some delay (τ_peak_) in the blue area of children’s LOS profile (see **Figure 4**), indicating a leading role of gestures on speech.

#### Research Question 2

The second research question is: what is the relative strength and direction of the interaction coupling between the gesture and speech subsystems? For this we looked at LAM, TT, and MaxL for both vertical and horizontal line structures, across the entire CRP. The mutual, ongoing, possibly asymmetric influence between gestures and speech will be visible in the CRP by the isentropic patterns of colored line structures representing same-tier skill levels. Accordingly, we expect vertical and horizontal LAM, TT and MaxL, and especially their differences, to inform us about the coupled dynamics of gestures and speech, and its potential asymmetry with regard to strength and direction.

#### Research Question 3

The third research question is closely related to the second, but focused on the specific skill-level tiers: what is the relative strength and direction of the interaction between gestures and speech *for the different levels of understanding* (i.e., skill-level tiers)? To investigate this, two CRPs were analyzed and compared for each child. The first CRP *only* displayed matches of gestures and speech of a skill level from the sensorimotor (S-)tier (i.e., levels 1, 2, or 3), while the second CRP *only* displayed matches of a skill level from the representational (R-)tier (i.e., levels 4, 5, or 6). Subsequently, vertical and horizontal LAM, TT, and MaxL were calculated from these CRPs, and compared on the group level. Furthermore, to capture the relative strength and direction of the coupling, that is, the asymmetry between gestures and speech within a child, we calculated a relative difference score for each measure, for each child. This relative difference score is defined as the standardized difference between the measures derived from the vertical lines minus the measures derived from the horizontal line, as follows: V-H_LAM_ was calculated as LAM_V_ – LAM_H_ (LAM is a proportion and can readily be compared), V-H_TT_ as (TT_V_ – TT_H_)/(TT_V_ + TT_H_), and V-H_MaxL_ as (MaxL_V_ – MaxL_H_)/(MaxL_V_ + MaxL_H_). A model simulation by Cox et al. (2016) of the relation between relative difference in coupling strength and relative difference in horizontal and vertical line measures showed a strong association between relative coupling strength and the difference between LAM and TT, but not for MaxL. The relative difference scores of the S- and R-tier scores were also compared on a group level.

There are two reasons to expect dynamic differences in the gesture–speech interaction for different levels of understanding. First, as explained, skill levels from the sensorimotor tier include expressions about perceptions, action, and observable relations between these perceptions and actions, whereas skill levels from the representational tier are assigned to expressions that go beyond these observable actions and perceptions. Previously, the link between gestures and cognition has been assigned to gestures adding action information to existing mental representations (Beilock and Goldin-Meadow, 2010) and gestures simulating actions (Hostetter and Alibali, 2010). This presumed close relation between actions and gestures might culminate in a different interplay between gestures and speech at the sensorimotor tier compared to the representational tier. Also, more complicated levels of understanding are likely to arise when the task is complicated, that is to say, when children perceive the task to be more challenging. A challenging task might trigger learning, and previously it has been shown that gesture–speech mismatches tend to occur when a child is on the verge of learning something new (Goldin-Meadow, 2003). As described earlier, we suggest that gesture–speech mismatches in a difficult, new and/or challenging task, arise from suboptimal simultaneous coordination of the gesture and speech synergies. When this suboptimal simultaneous coordination happens, the tight coupling between the action systems breaks down and becomes less dynamically stable and strong than for a less challenging task. Together we are inclined to expect that vertical and horizontal LAM, TT, and MaxL will show different patterns of values at different levels of understanding.

#### Research Question 4

The final research question is: how are the measures of coordination between gestures and speech subsystems related to more stable child characteristics and school outcome measures, such as age and general level of cognitive performance? Children’s use of speech and gestures is known to change over time (Goldin-Meadow, 1998). These changes are necessarily reflected in the dynamic organization of gestures and speech. Furthermore, as there is a link between gestures and cognition (Perry et al., 1988), children’s general level of cognitive performance is also expected to be related to this dynamic organization. We investigate these possible relations by calculating correlations between Age, Math score, Language score, and Average skill level across the previous five interactions with the researcher and the LOS-profile measures (%Sync, RR_peak_, Q_LOS_, and τ_peak_), the CRQA-measures (RR, LAM_V_, LAM_H_, TT_V_, TT_H_, MaxL_V_, and MaxL_H_) derived from the sensorimotor and representational tier, and the relative difference scores (V-H_LAM_, V-H_TT_, and V-H_MaxL_) for each of the tiers.

#### Monte Carlo Analysis

Throughout the Section Results, *p*-values for differences between two measures were calculated by using Monte Carlo permutation tests (Todman and Dugard, 2001), which enabled us to reliably obtain significance levels with this relatively small sample (Ninness et al., 2002). Using this procedure, the probability that an empirically observed difference can be found was repeatedly calculated, in this case 1000 times, each time using a random distribution of the original data. If the average probability that the difference occurs in these random samples was small (i.e., <0.05), we concluded that there is an actual difference present in the empirical data, which cannot be simulated using random samples, and hence, was not caused by chance. When a Monte Carlo permutation test was used to compare two values, we also calculated the effect size in the form of Cohen’s *d*, that is, the observed difference divided by the pooled SD. A value of *d* between 0.2 and 0.3 is generally considered to be small, a value around 0.5 as medium, and a value of 0.8 and higher as large (Cohen, 1988).

## Results

### Research Question 1: What is the Temporal Relation between Gestures and Speech, in Terms of their Displayed Skill Level?

For the first research question we expected that the LOS-profile analysis measures would display a significant asymmetry in the amount of recurrence around the LOS (Q_LOS_) and display a peak in the recurrence (RR_peak_) at some delay (τ_peak_), indicating a leading role of gestures on speech. An overview of the values for Q_LOS_, RR_peak_, and τ_peak_ in our sample can be found in **Table 2**. As described in the Section “Materials and Methods,” if Q_LOS_ is lower than 1, this suggests that gestures are leading speech in time. In our sample, Q_LOS_ ranged from 0.48 to 1.78, with an average of 1.08 which was not significantly higher than 1 (*p* = 0.72). The average Q_LOS_ (*M* = 0.86) of the children in Kindergarten was lower than the average Q_LOS_ (*M* = 1.24) of the children in first grade (*p* = 0.04, *d* = 0.90). This suggests that the gesture–speech dynamics had an opposite temporal pattern in the two age groups, with a leading role for speech for the first graders.

**Table 2:** Overview of LOS-profile measures and CRQA-measures of all 12 childrens.

		LOS profile analysis measures			CRQA-measures over entire CRP					
Child	Grade	Q_LOS_	RR_peak_	τ _peak_	RR	LAM_V_	LAM_H_	TT_V_	TT_H_	MaxL_V_	MaxL_H_
1	KG	0.46	0.056	18	0.013	0.986	0.910	5.2	3.4	21	7
2	KG	0.58	0.089	16	0.019	0.996	0.885	6.4	3.8	19	10
3	KG	0.91	0.015	–	0.004	0.968	0.687	4.3	2.6	12	3
4	KG	0.98	0.076	2	0.011	1.000	0.885	7.4	5.1	26	11
5	KG	1.31	0.012	36	0.002	0.893	0.901	3.2	3.1	5	6
6	1	1.28	0.034	-1	0.010	0.957	0.701	6.6	2.6	16	5
7	1	0.48	0.039	-1	0.009	0.979	0.922	5.8	4.0	18	12
8	1	1.65	0.034	0	0.006	0.973	0.624	4.8	2.8	12	5
9	1	0.90	0.140	0	0.025	0.992	0.924	6.3	5.1	15	15
10	1	0.92	0.053	-1	0.016	1.000	0.789	6.0	5.5	25	27
11	1	1.78	0.021	-1	0.002	0.959	0.632	5.4	2.7	18	3
12	1	1.66	0.073	-1	0.018	1.000	0.793	8.3	3.6	24	6
*Mean*	*–*	*1.08*	*0.053*	*6.09*	*0.011*	*0.975*	*0.805*	*5.8*	*3.7*	*17.6*	*9.2*

The observed RR_peak_ should exceed chance level, that is, there should be a real peak in the profile, for the observed τ_peak_ to make any sense. To verify this, a Monte Carlo procedure was performed to assess whether children’s observed RR_peak_ significantly differed from chance. This was the case for all children in our sample (all *p*-values < 0.01), except for child 3 (*p* = 0.63). Therefore τ_peak_ of child 3 was not included in the subsequent analyses of this research question. On average τ_peak_ was 6.09 within the group, which was significantly higher than 0 (*p* = 0.03), indicating that gestures were ahead of speech in time. The average τ_peak_ of children in Kindergarten (*M* = 18) differed from that of the first graders (*M* = -0.71; *p* < 0.01, *d* = 2.22). In addition, the average τ_peak_ of children in Kindergarten was significantly higher than 0 (*p* < 0.01) and the average τ_peak_ of children in the first grade was significantly lower than 0 (*p* < 0.01). This is conform the earlier result (above), meaning that for the younger children in our sample gestures were ahead in time of speech (18 s on average), whereas, oppositely, gestures were behind in time of speech (0.71 s on average) for the older children.

### Research Question 2: What is the Relative Strength and Direction of the Interaction between the Gesture and Speech Subsystems?

See **Table 2** for an overview of LAM, TT, and MaxL for both vertical and horizontal line structures. LAM_V_ ranged from 0.893 to 1.000 (*M* = 0.975), which means that 89.3–100% of the recurrent points comprised vertical line structures. TT_V_ ranged from 3.2 to 8.3 (*M* = 5.8), indicating that the average vertical lines in the recurrence plot consisted of 3.2–8.3 recurrent points. This reflects that gestures were trapped into same-tier skill-level episodes with average durations between 3 and 8 s for the different children. MaxL_V_ ranged from 5 to 26 (*M* = 17.6), which means that the maximum length of a vertical line in an individual recurrence plot ranged from 5 to 26 recurrent points. In other words, the maximum episode of gestures being trapped into a same-tier skill level lasted between 5 and 26 s. Calculations of the horizontal line structures revealed that the extent to which speech is trapped into displaying the same-tier skill level was somewhat less, with LAM_H_ ranging from 0.624 to 0.924 (*M* = 0.805), TT_H_ ranging from 2.3 to 5.5 (*M* = 3.7), and MaxL_H_ ranging from 3 to 27 (*M* = 9.2). At the group level, LAM_V_, TT_V_, and MaxL_V_ were higher than LAM_H_, TT_H_, and MaxL_H_, respectively (all *p*-values < 0.01; *d*_LAMV>_ _LAMH_ = 2.01; *d*_TTV__>__TTH_ = 1.72; *d*_MaxLV__>__MaxLH_ = 1.31). Interestingly, this is true for all children for LAM and TT, and for 9 out of 12 children also for MaxL. This finding clearly suggests an asymmetric dynamic attunement of gestures and speech, with gestures relatively more regularly and more rigidly displaying the same-tier skill level compared to speech.

### Research Question 3: What is the Relative Strength and Direction of the Gesture–Speech Interaction for Different Skill-Levels Tiers?

We expected RR and vertical and horizontal LAM, TT, and MaxL to be different for different levels of understanding. To analyze this, we first compared the averages of RR, LAM_V_, LAM_H_, TT_V_, TT_H_, MaxL_V_, and MaxL_H_ on the sensorimotor (S-)tier with those on the representational (R-)tier. An overview of these CRQA-measures can be found in **Table 3** (S-tier) and **Table 4** (R-tier). The differences between the CRQA-measures of the S-tier or R-tier are weak to absent (*p_RR_* = 0.19, *d* = 0.31; *p_LAM-V_* = 0.45, *d* = 0.05; *p_TT-V_* = 0.45, *d* = 0.03; *p_MaxL-V_* = 0.45, *d* = 0.05; *p_LAM-H_* = 0.42, *d* = 0.08; *p_TT-H_* = 0.91, *d* = 0.54; *p*_MaxL-H_ = 0.36, *d* = 0.12). This means that there were no group-level differences in the relative strength and direction of the interaction between gestures and speech for lower (S-tier) levels nor for higher (R-tier) levels of understanding.

**Table 3:** Overview of the CRQA-measures, calculated over skill levels 1–3 (sensorimotor tier).

	Child	Grade	% RR^∗^	LAM_V_	LAM_H_	V-H_LAM_	TT_V_	TT_H_	V-H_TT_	MaxL_V_	MaxL_H_	V-H_MaxL_
	1	KG	66.9	0.669	0.595	0.074	7.6	3.2	0.41	21	7	0.50
	2	KG	29.3	0.289	0.226	0.063	8.3	2.5	0.53	19	3	0.73
	3	KG	99.3	0.961	0.687	0.273	4.3	2.3	0.31	12	3	0.60
	4	KG	7.2	0.072	0.048	0.024	6.0	3.2	0.30	6	7	-0.08
	5	KG	73.3	0.733	0.672	0.061	3.2	3.1	0.01	5	6	-0.09
	6	1	95.6	0.915	0.672	0.243	6.7	2.6	0.44	16	5	0.52
	7	1	31.5	0.308	0.248	0.059	7.0	3.3	0.37	18	5	0.57
	8	1	73.9	0.721	0.480	0.241	4.3	2.7	0.24	8	4	0.33
	9	1	29.8	0.290	0.267	0.023	7.6	3.9	0.32	15	15	0.00
	10	1	60.3	0.603	0.539	0.064	5.1	5.4	-0.03	10	27	-0.46
	11	1	20.5	0.192	0.103	0.089	4.7	3.0	0.22	10	3	0.54
	12	1	19.8	0.198	0.161	0.037	5.0	3.3	0.21	9	5	0.29
*M*	*–*	*KG*	*55.2*	*0.545*	*0.446*	*0.10*	*5.9*	*2.9*	*0.31*	*12.6*	*5.2*	*0.33*
*M*	*–*	*1*	*47.3*	*0.461*	*0.353*	*0.11*	*5.8*	*3.4*	*0.25*	*12.3*	*9.1*	*0.26*
*M*		*Overall*	*50.6*	*0.496*	*0.391*	*0.104*	*5.8*	*3.2*	*0.28*	*12.4*	*7.5*	*0.29*

*^∗^% RR reflects the percentage of recurrence found on the S-tier, as compared to the overall recurrence rate on both the S- and R-tier, displayed in **Table 2***.

**Table 4:** Overview of the CRQA-measures, over skill levels 4–6 (representational tier).

	Child	Grade	% RR^∗^	LAM_V_	LAM_H_	V-H_LAM_	TT_V_	TT_H_	V-H_TT_	MaxL_V_	MaxL_H_	V-H_MaxL_
	1	KG	33.1	0.316	0.315	0.002	3.1	3.9	-0.11	5	7	-0.17
	2	KG	70.7	0.707	0.660	0.047	5.8	4.7	0.11	9	10	-0.05
	3	KG	0.7	0.007	0.000	0.007	3.0	0.0	1.00	3	1	0.50
	4	KG	92.8	0.928	0.837	0.090	7.5	5.3	0.17	26	11	0.41
	5	KG	26.7	0.160	0.229	-0.069	3.0	3.0	0.00	3	5	-0.25
	6	1	4.4	0.042	0.030	0.013	4.8	4.0	0.09	7	4	0.27
	7	1	68.5	0.671	0.674	-0.003	5.3	4.4	0.09	11	12	-0.04
	8	1	26.1	0.252	0.145	0.107	7.0	3.3	0.35	12	5	0.41
	9	1	70.2	0.702	0.657	0.045	5.8	5.9	-0.01	10	10	0.00
	10	1	39.7	0.397	0.250	0.147	8.2	5.7	0.18	25	8	0.52
	11	1	79.5	0.767	0.530	0.237	5.6	2.7	0.35	18	3	0.71
	12	1	80.2	0.802	0.632	0.170	9.8	3.7	0.45	24	6	0.60
*M*		*KG*	*44.8*	*0.424*	*0.408*	*0.02*	*4.5*	*3.4*	*0.23*	*9.2*	*6.8*	*0.09*
*M*	*–*	*1*	*52.7*	*0.519*	*0.417*	*0.10*	*6.7*	*4.2*	*0.21*	*15.3*	*6.9*	*0.35*
*M*	*Overall*	*Overall*	*49.4*	*0.479*	*0.413*	*0.066*	*5.8*	*3.9*	*0.22*	*12.8*	*6.8*	*0.24*

*^∗^% RR reflects the percentage of recurrence found on the S-tier, as compared to the overall recurrence rate on both the S- and R-tier, displayed in **Table 2***.

Next, we analyzed whether the measures derived from the vertical and horizontal line structures showed the same pattern of differences for the S-tier and R-tier. LAM_V_ was not higher than LAM_H_ for both the S-tier (*M_LAM-V_* = 0.496, *M_LAM-H_* = 0.391, *p* = 0.14, *d* = 0.38) and the R-tier (*M_LAM-V_* = 0.479, *M_LAM-H_* = 0.413, *p* = 0.30, *d* = 0.22). However, the analysis revealed TT_V_ to be higher than TT_H_ for both the S-tier (*M_TT-V_* = 5.81, *M_TT-H_* = 3.19, *p* < 0.01, *d* = 2.06) and R-tier (*M_TT-V_* = 5.75, *M_TT-H_* = 3.88, *p* = 0.01, *d* = 0.99). In addition, MaxL_V_ was higher than MaxL_H_ for both the S-tier (*M_MaxL-V_* = 12.42, *M_MaxL-H_* = 7.50, *p* = 0.03, *d* = 0.80) and R-tier (*M_MaxL-V_* = 12.75, *M_MaxL-H_* = 6.83, *p* = 0.02, *d* = 0.92). Lastly, the relative difference scores between the S-tier and R-tier did not differ (*p_V -H-LAM_* = 0.15, *d* = 0.43; *p_V -H-TT_* = 0.28, *d* = 0.22; *p_V -H-MaxL_* = 0.38, *d* = 0.13).

To summarize, the average differences between the CRQA-measures of vertical and horizontal lines showed the same pattern for the S-tier and R-tier. This means that the relative strength and direction of the coupling between gestures and speech did not differ between the levels of understanding. At the group level, they were similarly asymmetric for both tiers. Also, laminarity (LAM) did not show the same asymmetry at the individual levels of understanding, as it did when the tiers were joined together for Research Question 2.

#### Does Age Play a Role?

Prompted by the differences between younger and older children found for Research Question 1, we investigated whether similar age-group differences were present in the strength and direction of the interaction between gestures and speech for different levels of understanding. To this end, we compared the children in Kindergarten and first grade with regard to their CRQA-measures and relative difference scores on the S-tier and R-tier. These measures are displayed in **Tables 3** and **4**.

For the S-tier, no clear differences between the CRQA-measures of younger and older children were found (*p_RR_* = 0.26, *d* = 0.34; *p_LAM-V_* = 0.30, *d* = 0.26; *p_LAM-H_* = 0.25, *d* = 0.37; *p_TT-V_* = 0.46, *d* = 0.05; *p_TT-H_* = 0.07, *d* = 0.73; *p_MaxL-V_* = 0.50, *d* = 0.06; *p_MaxL-H_* = 0.12, *d* = 0.57). There were also no differences between the younger and older children with regard to the average relative difference scores on the S-tier (*p_V -HLAM_* = 0.41, *d* = 0.09; *p_V -HTT_* = 0.24, *d* = 0.36; *p_V -HMaxL_* = 0.35, *d* = 0.20). For the R-tier, only TT_V_ of the older children was higher than TT_V_ of the younger children (*p_TT-V_* = 0.04, *d* = 1.12). Even though the other CRQA measures on the R-tier might appear to be higher for the older children, no meaningful differences were found (*p_RR_* = 0.40, *d* = 0.17; *p_LAM-V_* = 0.31, *d* = 0.29; *p_LAM-H_* = 0.48, *d* = 0.03; *p_TT-H_* = 0.17, *d* = 0.54; *p_MaxL-V_* = 0.12, *d* = 0.73; *p_MaxL-H_* = 0.51, *d* = 0.02). Considering the relative difference scores, only V-H_LAM_ was higher for older than for younger children (*p_V -HLAM_* = 0.02, *d* = 1.11). There were no clear difference for V-H_TT_ (*p_V -HTT_* = 0.46, *d* = 0.06) and only slightly for V-H_MaxL_ (*p_V -HMaxL_* = 0.07, *d* = 0.85).

In conclusion, for the less difficult levels of understanding on the S-tier, older and younger children did not differ in the strength and direction of the interaction between gestures and speech. However, for the more difficult levels of understanding there were age-differences in the asymmetry of the gesture–speech interaction: gestures displayed longer average periods of lingering in the R-tier (TT_V_) and were more regular (V-H_LAM_) for the older children than for the younger children.

### Research Question 4: How are the Measures of Coordination between Gestures and Speech Subsystems Related to More Stable Child Characteristics and School Outcome Measures?

An overview of the significant correlations between child characteristics and school outcome measures, and the LOS-profile measures, CRQA -measures and relative difference scores can be found in **Table 5**. The entire correlation table is available in the supplementary materials. First we will describe the findings for the LOS-profile measures across both tiers, followed by the CRQA-measures and relative difference scores separately for each tier.

**Table 5:** Significant correlations between child characteristics and CRQA-measures.

		Age (months)	Math score	Language score	Average score past tasks
Both tiers	%Sync	0.57^∗^			
	τ_peak_	-0.73^∗∗^			
S-tier	LAM_V_		-0.54^∗^		-0.58^∗∗^
	LAM_H_				-0.52^∗^
	V-H_LAM_		-0.62^∗∗^		-0.58^∗∗^
	TT_V_			0.53^∗^	
	V-H_TT_			0.59^∗^	
R-tier	LAM_V_		0.51^∗^		0.56^∗^
	LAM_H_		0.57^∗^		0.54^∗^
	V-H_LAM_	0.65^∗∗^			
	TT_V_	0.51^∗^			
	TT_H_	0.61^∗∗^			0.61^∗∗^
	V-H_TT_		-0.68^∗∗^		-0.67^∗∗^
	MaxL_H_	0.65^∗∗^			
	V-H_MaxL_	0.52^∗^	-0.50^∗^		

*Values marked with ^∗^ are significant at p < 0.1, values marked with ^∗∗^ are significant at p < 0.05. The complete correlation matrix can be found in the supplementary materials.*

When recurrences on the sensorimotor and representational tier are combined, the correlation of %Sync and age had a value of 0.57. This means that relatively older children tended to show the same-tier skill level at the same time in gestures and speech. The correlation of -0.73 between τ_peak_ and age in months corroborates to this finding, as it implies that younger children tended to show a more extensive delay between gestures and speech in displaying the same-tier skill level, with gestures being ahead of speech in time.

For the S-tier separately, LAM_V_ and V-H_LAM_ were both negatively correlated with children’s Math score and Average score on past tasks (*r* = -0.54 and *r* = -0.58, respectively). This means that for children who performed better on math and past tasks, gestures were being trapped into S-tier episodes less prominently. Moreover, for these children the asymmetry between gestures and speech was smaller. LAM_H_ was also negatively correlated with the average score on past tasks (*r* = -0.52), which suggests that for children with a higher score on past tasks, speech was less prone to be trapped into S-tier episodes as well. Language score was correlated with TT_V_ (*r* = 0.53) and V-H_TT_ (*r* = 0.59) on the S-tier, which shows that for children with a higher Language score, gestures were trapped into longer average S-tier episodes, and that the associated asymmetry between gestures and speech tends to be bigger.

For the more difficult skill-levels on the R-tier, it turns out that all CRQA and LOS profile measures are significantly correlated with age or measures of general performance. Both LAM_V_ and LAM_H_ are correlated with Math score (*r* = 0.51 and *r* = 0.57, respectively) and the average score on past tasks (*r* = 0.56 and *r* = 0.54, respectively). This suggests that for children with a higher score on math or past tasks, both speech and gestures were trapped into R-tier episodes more often. Age correlates with V-H_LAM_, which means that the asymmetry between gestures and speech tended to be bigger for older children. TT_V_ was related to Age (*r* = 0.51), suggesting that older children were trapped into longer average R-tier gesturing episodes. Both Age and Average score on past tasks were correlated with TT_H_ (*r* = 0.61 and *r* = 0.61, respectively), which means that children who are older or who performed better on past tasks were trapped into longer average R-tier speech episodes. As V-H_TT_ is negatively correlated with both Math score and Average score on past tasks (*r* = -0.68 and *r* = -0.67, respectively), children who performed well on math or past tasks tended to display a smaller asymmetry in the average duration of gestures and speech R-tier lingering. MaxL_H_ and V-H_MaxL_ were related to age (*r* = 0.65 and *r* = 0.52, respectively), which suggests that older children had a longer maximum episode of speech being trapped at the R-tier, but at the same time, the asymmetry between gestures and speech tended to be larger for this. Finally, V-H_MaxL_ was negatively correlated with Math score (*r* = -0.50). So children with a higher score on math had a smaller asymmetry in the longest gestures and speech R-tier lingering episode.

## Discussion

### Summary of Results

The present study concentrated on how the earlier reported leading role of gestures over speech in children’s cognitive change arises from the asymmetries in the dynamic attunement of gestures and speech during task performance. Appreciating the dynamic nature of this issue naturally implied using of the language and methods of complex dynamical systems. Accordingly, we used CRQA, a novel non-linear time series method, to analyze the two skill-level time series as coded from children’s gestures and speech while they were working on an educational science task. To be able to address this rather broad issue intelligibly we proposed four specific research questions, focusing on: (1) the temporal relation between gestures and speech, (2) the relative strength and direction of the interaction between gestures and speech, (3) the relative strength and direction between gestures and speech for different levels of understanding, and (4) the relations between measures of dynamic organization and more stable child characteristics and school outcome measures.

Firstly, regarding the temporal relation, older and younger children differed in the (temporal) asymmetry in the gestures–speech interaction. In the 2 min window of the LOS-profile analysis, in younger, i.e., Kindergarten, children, the balance leant more toward gestures leading speech in time, whereas the balance leant more toward speech leading gestures in time for the older first-grade students. This difference between older and younger children is even more pronounced when we look at the actual temporal delay in seconds. While gestures are, on average, ahead of speech for 18 s for the younger children, speech only slightly precedes gestures for just under a second for the older children.

Secondly, we investigated the relative strength and direction of the interaction between gestures and speech as it plays out on all possible timescales, ranging from the sample rate (1 s) to the entire interaction (∼489 s). As described earlier, calculating and comparing recurrence measures of vertical and horizontal line structures is informative about the coordinative structures in the gesturing–speech interaction. At the group level, we found LAM, TT, and MaxL to point toward speech influencing gestures more regularly and rigidly into displaying the same-tier skill level than vice versa. Moreover, when comparing the strength and direction for different levels of understanding (Research Question 3), this asymmetry in gestures and speech extended to both the sensorimotor and representational tier. The relative difference scores did not differ for the S-tier and R-tier. In other words, there are no differences in the coupling between gestures and speech for different levels of understanding at the group level.

However, when we compared the CRQA measures for different levels of understanding of children from first grade and Kindergarten, an interesting pattern of differences appeared. Although no differences were present at the S-tier, at the more difficult R-tier level of understanding, older and younger children did differ in the coupling between gestures and speech. All CRQA measures were higher for the older children at the R-tier, suggesting that the coupling between gestures and speech was more rigid at higher levels of understanding.

The relation of age with the coupling between gestures and speech is also apparent when we relate the CRQA measures to individual child characteristics. The correlations between age and %Sync, and between age and τ_peak_ support the results from the LOS-profile analysis. This again shows that gestures are more ahead of speech in time when children are younger, and that they are more temporally aligned when children are older. The results reveal a larger asymmetry in the gesture–speech attunement for older children. A higher score on schools’ standardized language tests is also related to more asymmetry between gestures and speech, but only for the less difficult levels of understanding (S-tier).

However, children’s average score on past tasks and their scores on math seem to be related to speech attracting gestures less, and also to less asymmetry between gestures and speech for the less difficult levels of understanding. For the more difficult levels of understanding (R-tier), both speech and gestures tend to attract each other more for children with a higher score on math or past tasks, which points to more symmetry between speech and gestures. Moreover, a higher score on math or past tasks is also related to less asymmetry between gestures and speech at the R-tier.

### Dynamic, Entangled Development of Gestures, Speech, and Cognitive Skills

Earlier studies have shown that children express new cognitive insights by means of gestures before they are able to put them into words. An important nuance following from the present study is that although gestures might appear to be ahead in time of speech during children’s learning, this does not imply that gestures influence speech to a larger extent. Learning is a process that occurs at multiple, nested time scales, by means of entangled processes of action, perception and cognition. In studies thus far, such a process approach has not been considered with respect to the interplay of gestures and speech in children’s learning. At the very least our study shows that the relation between gestures, speech, and cognition in our sample is much more dynamic and bidirectional than previously thought, with a high degree of inter-individual variability. In addition, children differ in how speech and gestures are coupled, whereby gestures are not always ahead of speech, or leading speech, as cognitive understanding unfolds. Moreover, the gestures-speech coupling is related to age and measures of scholastic and cognitive performance that exceed the time-span of a single task.

#### Age, Language Score, and the Dynamic Emergence of Speech and Gestures

One particularly prominent result is that, with increasing age, speech and gestures become more synchronized and tightly coupled. Within this tight coupling for older children, speech attracts gestures more than vice versa in displaying the same-tier skill level. A possible explanation for this finding can be found in Iverson and Thelen’s (1999) account of the dynamic emergence of speech and gestures. They suggest that the link between speech and gestures starts with the hand-mouth linkage that is already apparent in newborns. Coordination between oral and manual actions is very common in newborn’s spontaneous actions, such as bringing their hands to the facial area or sucking their fingers. These connections between oral and manual actions are characterized by a low threshold—as they are so easily and spontaneously performed—and high activation, because of their frequency. Around the age of 6–8 months, both rhythmical arm movement and rhythmical babbling emerge, through which coinciding vocal and manual activities are entrained.

The linkages between the hands and mouth become more controlled as children develop, with the emergence of the first gestures and words around 9–14 months of age. Typically, children’s gestures precede and outnumber their spoken words tremendously during this period. To be more specific, children’s pointing gestures precede the word for an object by, on average, 3 months, and gesture-plus-word combinations precede two-word combinations by an average of 4.7 months (Iverson and Goldin-Meadow, 2005). According to Iverson and Thelen (1999), the reason for this is that, in comparison to the vocal articulators, the control of the hands is more advanced and therefore it is easier for children to communicate by means of gestures. In other words, for gestures the threshold is low and activation is high, while for speech the threshold is high and activation is low. However, as children practice their vocal skills, the threshold of speech becomes lower and activation higher. The activation of speech eventually becomes so high, that it captures and concurrently activates gestures. Stated differently, as children’s language skills become more advanced, their speech system activates their gesture system, and thereby the two motor systems become more synchronized.

Returning to our finding that older children in our sample show higher levels of synchronization and coupling between speech and gestures. It is safe to assume that older children have more developed speech and gesture synergetic control. The reason for this is that both action systems have been explored and practiced more, and under more different and variable task conditions, than in the younger children (cf. Iverson and Thelen, 1999). Because of this, the speech and gesture synergies are more entrained, which means that older children can coordinate the two synergies more optimally and simultaneously. This reasoning and the finding that speech influences gestures more than vice versa in older children, is in line with Iverson and Thelen’s (1999) notion of speech capturing gestures when vocal skills become more practiced. A final noteworthy observation in this respect is that the older children in our sample just entered first grade, in which they learn to read and write. Although speculative at this point, it is not farfetched to expect that this emphasis on language in the first grade increases how much speech is able to influence gestures (cf. Shanahan and Roof, 2013).

As already implied in the previous section, the explanation that gestures are ahead of speech in time for the younger children, with an average delay of 18 s, might also be found in the simultaneous coordination of the synergies of speech and gestures. For the younger children the task might be more difficult than for the older children, and pose considerably more conflicting task constraints. These conflicting task constraints may cause the two synergies to be unable to simultaneously exist in an optimal way. This makes the tightly coupled synergies dissociate, with the gesture synergy being created first and the speech-synergy later. The average lag of 18 s between speech and gestures might intuitively seem hard to understand, but such contingencies over relatively large timescales have been found before in the context of communication, albeit with younger children. For example, Jaffe et al. (2001) report a 20–30 s lag between contingencies in the vocal patterns of 4-months-old infants and their mothers or strangers. Moreover, Jaffe et al. (2001) point to other studies, which found a 20–30 s cycle in infant attention (Brazelton et al., 1974), a 10–45 s cycle in coordination of facial engagement (Lester et al., 1985), and a 20 s lag in facial engagement correlation (Cohn and Tronick, 1988). Although this concerns interpersonal coordination, these studies demonstrate that latencies of this magnitude are not extreme.

Jaffe et al. (2001) propose that the 20–30 s lag between contingencies in the vocal patterns of the infants and their mother or a stranger is an indication for a slow rhythm in the interaction. This slow rhythm can only be found by analyzing the data in much detail, as opposed to rhythms such as vocalization-pause or turn taking, which are detectable for untrained observers. To return to our study: both speech and gestures are suggested to originate from coinciding rhythmical activities (Iverson and Thelen, 1999; Abney et al., 2014), and in fact, speech and gestures are rhythmical activities in itself (Loehr, 2007). The average delay between speech and gestures of 18 s that we found for the younger children might be a slow rhythm in the gesture–speech interaction. This slow rhythm may reside in a process on a larger timescale, in which both the synergies of gestures and speech are nested. Which specific process this would be remains a question for future research.

The relation we found between a higher language score and more asymmetry in the speech-gesture coupling fits with the dynamic emergence account of speech and gestures as outlined above. With an explained variance of 25%, better language skills are associated with a stronger influence of speech on gestures. Interestingly, the relation between a higher language score and more asymmetry is not apparent for the higher levels of understanding on the R-tier. An explanation for this might be that the levels of understanding on the R-tier go beyond the skill of naming observable task characteristics, but rather involve relations among task elements, and relations among relations (cf. Fischer and Bidell, 2006; see below). A second explanation might be that understanding on the R-tier is more difficult, which causes a different interplay between the synergies of speech and gestures than on the S-tier - in this case less asymmetry in influence.

#### Average Score on Past Tasks, Math-Score, and Higher-Order Understanding Emerging from Actions

Contrary to age and language score, a higher average score on past tasks is not related to a leading role of speech over gestures, but instead to a more symmetric interaction between speech and gestures. In order to grasp this finding, consider how higher-order understanding emerges from actions. In a previous study, participants were asked to perform a gear task and predict the turning direction of a target gear (Trudeau and Dixon, 2007). At first, all participants simulated the motions of the gears with their hands, i.e., force tracing, to predict in which direction the target gear would turn. After a certain number of these problems, participants discovered a higher-order relation, alternation, which is concealed in the task. Alternation, like all higher-order relations, is a relation among relations and requires coordinating two or more lower-order relations and integrating multiple actions over time. Participants varied considerably in how many simulations they performed before discovering alternation, and Trudeau and Dixon (2007) found that the number of alternating actions performed before discovering the higher-order rule predicted the likelihood of generalization of this rule to new problem types. Trudeau and Dixon (2007) explained this finding by stating that for participants who made more correct alternating actions before discovering the higher order rule, the representation of alternation stems from a larger corpus of actions. This larger corpus of relevant (i.e., task-related) actions increases the chance of discovering and being able to generalize the higher-order relation. Extrapolating on this thought, children for whom speech is less leading over gestures might be more open to gesturing, that is, they might gesture more. This provides them with the larger corpus of actions, which increases their chance of discovering higher-order relations by means of actions, resulting in a higher score on (past) tasks.

Even more so, the gestures of children may also elicit discovering these higher-order relations in other tasks. Indeed, Smith (2010) has emphasized the essence of the motor system in learning higher-order regularities. She states that “It is action that creates a task, that couples component systems in the moment, and that selects and creates the momentary dynamic input on which learning must depend” (p. 264). In the context of action, component systems become coupled and coordinated within diverse tasks, which makes action essential for learning higher-order relations and generalizing learning such relations to other tasks. With respect to our findings, gesturing may also elicit the discovery of higher-order relations in other tasks, which might explain why children for whom speech was less leading over gestures performed better on past tasks.

Next to a higher average score on past tasks, a higher math score is also related to a more symmetric interaction between gestures and speech, whereby speech is less leading over gestures. Note that these two scores were highly correlated (*r* = 0.84), meaning that children who scored high on math were also likely to have done well on previous tasks. It is well-known that gestures are beneficial for math learning (e.g., Cook and Goldin-Meadow, 2006; Ehrlich et al., 2006; Broaders et al., 2007; Alibali and Nathan, 2012; Cook et al., 2012, 2013; Novack et al., 2014). The reason why gestures are related to math might be the same as why gestures are related to a higher average score on past tasks: from gestures, higher-order (mathematical) understanding can emerge and generalize. Indeed, Cook et al. (2013) and Novack et al. (2014) found that gestures are related to the generalization and transfer of mathematical knowledge to new problem types.

To summarize this subsection, children within our study for whom speech is less leading over gestures may perform better on both math and past tasks because they are more open to gesturing, from which higher-order understanding is thought to emerge and generalize. A reason for this might lie in the importance of variability in learning (e.g., Van Geert and Van Dijk, 2002). If the first system influences the second system to a lesser degree, that second system is obviously less constrained by the first, and can adhere more adaptively to task requirements. In other words, the coupling of the two systems can be characterized as more flexible, which allows for different types of coordination between them and with the environment.

Following the framework introduced earlier, we interpret the finding that speech is less leading over gestures in terms of synergetic competition. Accordingly, a decreasing leading role of speech over gestures indicates a more optimal and efficient (simultaneous) coordination of both synergies. However this optimal coordination of both synergies does not necessarily have to be simultaneous since we found that more temporal synchrony of speech and gestures is not related to a better score on past tasks or a better math performance in our study. Future studies could focus more specifically on how improved understanding of concepts and/or performance on a task is related to a more optimal (possibly but not necessarily simultaneous) coordination of both the speech and gestures synergies. This could become visible, for instance, by a decrease in the temporal delay between gesture and speech behaviors (Goldin-Meadow et al., 1993), a phase-transition like change from a period of suboptimal coordination of one or both synergies to state of simultaneous optimal coordination (cf. Haken et al., 1985), or perhaps by a change in the temporal structure of gestures and speech (Wijnants et al., 2009, 2012a; cf. Den Hartigh et al., 2015).

## Conclusion

Our results suggest that speech and gestures may be more *tightly coupled* for the older children in first grade and children with a high language score, because their speech and gesture systems are more developed. The reason that speech *leads* over gestures for these children may as well stem from this developmental process, and might be enhanced by the emphasis on language in first grade. For children with a higher average score on past tasks and math score, speech is leading less over gestures, possibly leaving more room for higher-order understanding to emerge from their action experiences through gesturing. Because of the time-intensive coding procedures and the in-debt nature of our analyses, this study used a small *N*. Note that we used Monte Carlo permutation tests, which are particularly strong in the case of small sample sizes. The credibility of our results is further strengthened by the relatively large effect sizes we found (Cohen, 1988). Nonetheless, this study deserves replication to check whether the findings can be verified and eventually also further refined and strengthened.

It is important to note that speech leading less over gestures is not the same as *less speech* or *less coupling between gestures and speech*. In fact, higher-order understanding, and more broadly speaking, cognition itself, resides in and emerges from the coupling between a multitude of perception-action subsystems, such as those related to speech and gestures (Goldin-Meadow, 1998). Congruously, within our study, the child for whom there was no RR_peak_, that is, for whom coupling between gestures and speech was weaker, had low scores on all the other variables of cognitive performance. To elaborate, it is not the mere presence or absence of coupling between subsystems that is important, but rather the nature of their coupling, in the sense of interaction-dominant dynamics (Van Orden et al., 2003, 2005). How the subsystems are coupled determines how development will progress, and whether and how higher-order understanding will occur. Our findings suggest that a coupling in which the influence of gestures and speech is more balanced (i.e., where speech is less leading), seems to be beneficial for higher-order understanding to develop in this respect, in a hands-on science and technology task.

As cognition resides in and emerges from the dynamic coupling between perception-action subsystems, and learning is a non-linear process with variability as its hallmark, methods that capture this coupling over time are necessary to understand how development comes about. The complex dynamical systems approach provides a framework for asking question and interpreting answers pertaining to how higher-order relations can emerge from perception-action couplings. In our study, we investigated how the speech and gesture subsystems of children are coupled during a hands-on educational science task. Among other things, we found this coupling to be related to other measures of cognitive performance. Instead of gestures expressing or adding to a rather disembodied cognitive insight before speech is able to express it, we outlined how higher-order understanding might emerge from the changing coupling between gestures and speech over time. Moreover, we proposed a new mechanism, of competing and suboptimal coordinated synergies resulting in gestures-speech mismatches, that builds a bridge between the existing research on gestures and recent views on cognition as fundamentally embedded and embodied. Future studies should investigate if the dynamic organization of gestures and speech indeed points to gesture–speech mismatches as originating from competing synergies of gestures and speech.

## Author Contributions

LDJ-H: draft of manuscript, coding of videos, design coding system gestures, design figures and tables, interpretation of analyses. SVdS: draft of manuscript, task administration with children, coding of videos, design coding system speech, correlation analyses. PVG: editing of manuscript, general advice on research procedure and writing, suggestions for references, calculation effect sizes. RC: draft and editing of manuscript, recurrence quantification analyses (ideas and execution), interpretation of analyses.

## Conflict of Interest Statement

The authors declare that the research was conducted in the absence of any commercial or financial relationships that could be construed as a potential conflict of interest.
