# Supplementary material for: Asymmetric Dynamic Attunement of Speech and Gestures in the Construction of Children’s Understanding
Source: Front Psychol. 2016 Mar 31;7:473. doi: 10.3389/fpsyg.2016.00473 (PMC4814764; doi:10.3389/fpsyg.2016.00473)
Supplement: Supplementary file 3 [file Data_Sheet_3.PDF]

Appendix: Coding procedure

Coding of verbal expressions

All children's verbal expressions were coded in four steps using the computer program MediaCoder (Bos & Steenbeek, 2006). First, we started with the determination of the exact points in time when utterances of children started and ended. Then we classified the verbal utterances into six categories: Descriptive, predictive, and explanatory utterances; requests; content-related questions, and miscellaneous.

After these two steps, meaningful units of the children's coherent descriptive, predictive, and explanatory utterances were formed, the so-called 'units'. Each utterance corresponded to one unit. However, when two or more utterances only had a short pause in between (< 2 s), and focused on the very same topic, we also considered this as one unit, which meant that we could group them together in the next step of the coding process (see below). Each unit ended when the next expression of the child fell into another category, when there was a longer pause between the child's utterances, or when the researcher interrupted the child (e.g., by asking another question, or by making a procedural remark). An exception was made for short and simple expressions of encouragement of the researcher (e.g., " I see").

In the fourth step, the complexity of the utterances within a unit was determined. This meant that each unit was rated on a scale based on the model of dynamic skill theory developed by Fischer (1980). At Level 1 (sensorimotor actions), children stated single characteristics of the task, such as "This tube is long". At Level 2 (sensorimotor mappings), two elements of the task were coupled, such as "I can push this [piston] into here [pump]". At Level 3 (sensorimotor systems), simple causal mechanisms were stated, such as "If I push this [pump] in, the balloon grows bigger". At Level 4 (single representations), two causal mechanisms were coupled, or an "invisible" causal mechanism was mentioned, such as "When I push this [pump], *air* travels to the balloon". Explanations involving two causal relationships and an additional step were classified at Level 5 (representational mappings),

e.g. “The piston pushes the air down, which goes through the tube to the other syringe, which piston then gets pushed out by the air”. Level 6 (representational systems) comprised utterances in which all relevant representations that play a role within the task are mentioned. Level 7 comprised abstract utterances, for example about air pressure, or compression. Level 1-3 are part of the sensorimotor tier, level 4-6 are part of the representational tier, and level 7 is part of the abstract tier. In the original theory, 3 more levels are specified, but these develop at later ages and were therefore not specified for this study. Incorrect, irrelevant, and “don’t know”-answers were rated as incorrect.[1]

The questions and units of answers received a code on an ordinal scale from 1 to 7 (ranging from sensorimotor actions to single abstractions). The coding 0 was used to mark the end of each utterance. Only utterances that displayed correct characteristics or possible task operations or mechanisms were coded as a skill level.

Coding of gestures

Gestures and task manipulations were coded in three steps. First, we coded the exact points in time when gestures and task manipulations of children started and ended. In this step we also noted whether the gesture could be characterized as 1) a short answer (short, task-related gestures, usually serving as an answer to a question), 2) a representation of the task or a task manipulation, or 3) an emblem. The latter category comprised task-*un*related short gestures with a rather universal character (e.g., ‘thumbs up’), which were not subject to further analysis.

In the second step, we further classified the categories short answers and representations/manipulations. Short answers were classified into: Nodding yes, shaking the head (“no”), lifting both shoulders (“don’t know”), and pointing toward (part of) the task. The representations/manipulations were further classified into: Representing a *characteristic* of the task, representing a *movement* of (elements of) the task, representing a *relationship* between two or more task elements, representing an *abstraction*, *single manipulations*

---

[1] For earlier use and more examples of this scale, see Van Der Steen, Steenbeek, Wielinski, & Van Geert, 2012; Van Der Steen, Steenbeek, & Van Geert, 2012; see also Rappolt-Schlichtmann, Tenenbaum, Koepke, & Fischer, 2007 for another application of this theory.

(simple procedural manipulations of the task, e.g., pushing the syringe, turning a tap), and miscellaneous.

In the last step, we classified the short answers into 'right', 'wrong', or 'other', and we assigned skill levels to the representations/manipulations, again based on Skill Theory (Fischer, 1980; Fischer & Bidell, 2006). At Level 1 (sensorimotor actions), the child described (in gestures) a single characteristic of the task or an object that was directly observable (e.g., stating that something is heavy, soft, hard, small, etc.). At level 2 (sensorimotor mappings), the gestures of the child represented simple, observable relationships between elements of the task, for example, a gesture that depicts a simple direction of movement. At level 3 (sensorimotor systems), gestures depicted observable causal relationships between elements, such as describing two subsequent movements, or gestures depicting a cause and effect. Level 4 (single representations) comprised gestures not involving direct observable elements, such as when the child made a prediction, or gestured about invisible mechanisms (air), or when he or she connected two causal relationships. Level 5 (representational mappings) is assigned when the child's gestures connect two or more single representations, such as correctly predicting (single representation 1) the flow of air (representation 2) within the task. Level 6 (representational systems) covers gestures in which all relevant representations that play a role within the task are mentioned. Finally, we scored level 7 when the gesture contained an abstraction, such as a representation of air compression. Incorrect, irrelevant, and "don't know"-answers were rated as incorrect.

The gestures received a code on an ordinal scale from 1 to 7 (ranging from sensorimotor actions to single abstractions). The coding 0 was used to mark the end of each utterance, and for utterances. Only utterances that displayed correct characteristics or possible task operations or mechanisms were coded as a skill level.
