# Supplementary figures and images for: Asymmetric Dynamic Attunement of Speech and Gestures in the Construction of Children’s Understanding
Source: Front Psychol. 2016 Mar 31;7:473. doi: 10.3389/fpsyg.2016.00473 (PMC4814764; doi:10.3389/fpsyg.2016.00473)

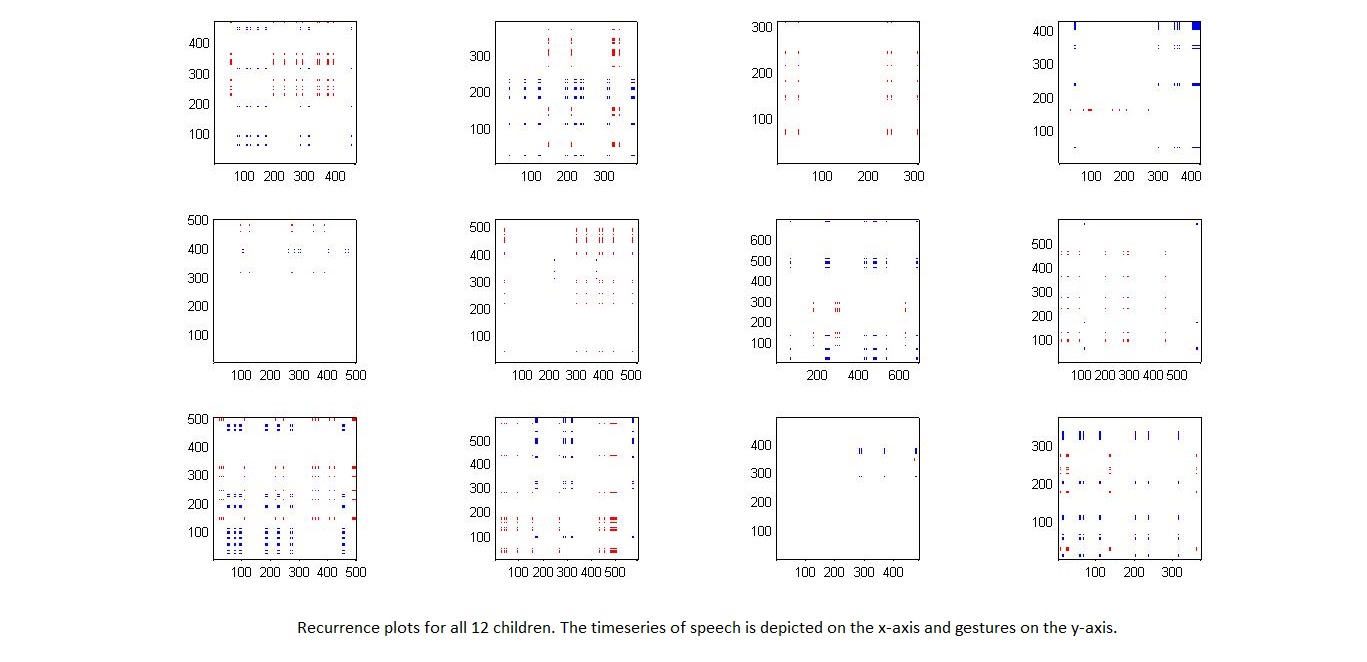

Supplement: Supplementary file 4 [file Image_1.JPEG]
